# Non‐invasive prenatal diagnosis of Duchenne and Becker muscular dystrophies by relative haplotype dosage†

**DOI:** 10.1002/pd.4781

**Published:** 2016-02-23

**Authors:** Michael Parks, Samantha Court, Siobhan Cleary, Samuel Clokie, Julie Hewitt, Denise Williams, Trevor Cole, Fiona MacDonald, Mike Griffiths, Stephanie Allen

**Affiliations:** ^1^ West Midlands Regional Genetics LaboratoryBirmingham Women's NHS Foundation TrustBirminghamUK

## Abstract

**Objective:**

Development of an accurate and affordable test for the non‐invasive prenatal diagnosis of Duchenne and Becker muscular dystrophies (DMD/BMD) to implement in clinical practice.

**Method:**

Cell‐free DNA was extracted from maternal blood and prepared for massively parallel sequencing on an Illumina MiSeq by targeted capture enrichment of single nucleotide polymorphisms (SNPs) across the dystrophin gene on chromosome X. Sequencing data were analysed by relative haplotype dosage.

**Results:**

Seven healthy pregnant donors and two pregnant DMD carriers all bearing a male fetus were recruited through the non‐invasive prenatal diagnosis for single gene disorders study. Non‐invasive prenatal diagnosis testing was conducted by relative haplotype dosage analysis for X‐linked disorders where the genomic DNA from the chorionic villus sampling (for healthy pregnant donors) or from the proband (for pregnant DMD carriers) was used to identify the reference haplotype. Results for all patients showed a test accuracy of 100%, when the calculated fetal fraction was >4% and correlated with known outcomes. A recombination event was also detected in a DMD patient.

**Conclusion:**

Our new test for NIPD of DMD/BMD has been shown to be accurate and reliable during initial stages of validation. It is also feasible for implementation into clinical service. © 2016 The Authors. *Prenatal Diagnosis* published by John Wiley & Sons, Ltd.

## Abstract 2

**What's Already Known About This Topic?**

- Recent research has shown that non‐invasive prenatal diagnosis for some single gene disorders is possible, and there is some implementation into clinical practice for the detection or exclusion of the paternal allele. However, this is not yet available for definitive diagnosis of X‐linked conditions.

**What Does This Study Add?**

- We have developed an accurate and feasible test for the non‐invasive prenatal diagnosis of Duchenne and Becker muscular dystrophies. Early validation data have proven that the method could potentially be implemented into clinical practice.

## Introduction

Since the discovery of cell‐free fetal DNA (cffDNA) in maternal plasma during pregnancy,^1^ many advances have been made in the research for highly sensitive and reliable non‐invasive prenatal diagnostic (NIPD) tests.^2^ cffDNA is composed of small fragments of extracellular DNA derived from the shedding of placental trophoblasts^3^ and only accounts for around 10% of cell‐free DNA (cfDNA) circulating in the maternal bloodstream.^4^, ^5^ Therefore, the use of cffDNA in clinical applications has been limited to the detection of paternally inherited sequences^6^, ^7^, ^8^, ^9^, ^10^ and *de novo* mutations.^11^ However, recent technological breakthroughs in the field of massively parallel sequencing (MPS) have enabled the development of clinical tests aimed at detecting fetal aneuploidies at early gestational age.^2^, ^12^, ^13^, ^14^, ^15^ Further research has also been conducted with the aim of developing NIPD tests for single gene disorders (SGDs).^16^, ^17^ Various proofs of principle studies have been published on NIPD testing of β‐thalassemia,^18^, ^19^ congenital adrenal hyperplasia (CAH)^20^, ^21^ and Duchenne and Becker muscular dystrophies (DMD/BMD)^22^ using MPS. However, these tests have not yet been translated into clinical practice because of the elevated costs of high‐throughput MPS.

As part of the non‐invasive prenatal diagnosis for single gene disorders (NIPSIGEN) project conducted at Birmingham Women's National Health Service (NHS) Foundation Trust (UK), we aimed at developing an affordable NIPD test for SGDs. After carefully considering various methods described in previous studies,^18^, ^23^ we decided to adopt the relative haplotype dosage (RHDO) analysis developed by Lo and colleagues.^24^ In 2010, Lo was able to construct a genome‐wide genetic map of the fetus from maternal plasma DNA sequences using RHDO^24^ and subsequently demonstrated how this could be applied for NIPD of β‐thalassemia^19^ and CAH.^20^ In a similar manner, we were able to apply RHDO analysis for the non‐invasive prenatal detection of DMD/BMD disorders in at risk pregnancies. Moreover, by using a highly targeted and efficient enrichment process, our method allows for multiplexing of several patients on a single sequencing run of an Illumina MiSeq. This makes our test feasible from a clinical perspective.

Duchenne and Becker muscular dystrophies are X‐linked neuromuscular recessive disorders associated with mutations of the dystrophin gene.^25^ DMD is the most common of the two with an incidence of 1:3500 male newborns, while BMD has a lower incidence of 3:100 000 male newborns and presents a milder clinical course and slower disease progression compared with DMD.^26^, ^27^ The mutational profile of DMD/BMD is extremely varied, with 60–65% of mutations caused by large deletions within the dystrophin gene; 5–10% by partial gene duplications; and the remaining 25–30% by small mutations.^26^ The current practice in prenatal diagnosis for women with a fetus at risk of DMD/BMD is to offer non‐invasive fetal sexing^9^ and, if the fetus is male, to analyse fetal DNA obtained by invasive procedures, such as chorionic villus sampling (CVS) and amniocentesis, to assess the mutational profile of the dystrophin gene.^28^ Invasive procedures are associated with a 0.5–1% risk of miscarriage,^29^, ^30^ and no alternative is currently available for women with a male pregnancy at risk of DMD/BMD who decline their use. The introduction of NIPD tests for SGDs would provide a viable alternative to invasive procedures with the additional benefits of no miscarriage risk and testing at early gestational age. Our method for NIPD of DMD/BMD has shown promising results on patients tested so far and has the potential to be implemented into clinical service.

## Materials and Methods

### Patient groups and sample workflow

**Figure 1:**
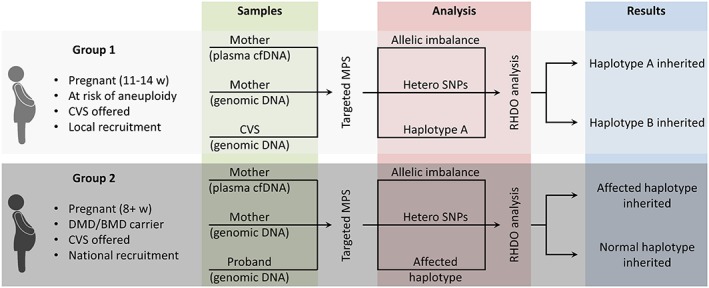
Workflow and processing steps of samples obtained from group 1 and group 2 patients. As group 1 patients consisted of individuals who were not at risk of Duchenne and Becker muscular dystrophies pregnancy, the final outcomes of the test were labelled as simply haplotype A or B

Patients were recruited into two separate groups through the NIPSIGEN study (‘NIPSIGEN: clinical translation of NIPD for SGDs’; REC approval number: 13/NW/0580). Group 1 included pregnant women at risk for fetal aneuploidy who were offered invasive prenatal testing (CVS) at West Midlands Regional Genetics Laboratory. Blood samples from women with male pregnancies in this group were initially used to validate the efficiency, accuracy and multiplexing capacity of our method. RHDO analysis can be performed on these patients by using the genomic DNA from CVS to determine the reference haplotype needed to measure the allelic imbalance within the plasma cfDNA (Figure 1). Pregnant women who are known carriers of a DMD/BMD mutation were recruited nationwide (UK) to group 2. For these patients, we requested a sample of genomic DNA from a previously affected child (or other affected relative) to use in determining the affected haplotype (Figure 1). The DNA samples needed for each patient included the cfDNA extracted from maternal plasma; the maternal genomic DNA extracted from leukocytes; and the proband genomic DNA (from the CVS sample for group 1 patients and from a previous affected relative for group 2 patients) (Figure 1). All three samples from a maximum of three patients are processed simultaneously and are pooled together prior to targeted capture enrichment and MPS. cfDNA was extracted from 4 ml of plasma and eluted in a final volume of 60 µl. Maternal genomic DNA was extracted from the leukocytes contained in 1 ml of the blood cell portion. Details on sample processing and DNA extraction can be found in Appendix A in the supporting information. After targeted MPS, sequencing data from each DNA sample were used to perform RHDO analysis and determine fetal inheritance of the dystrophin gene. The outcomes were set as ‘haplotype A’ and ‘haplotype B’ for group 1 patients; and ‘affected’ and ‘unaffected’ haplotype for group 2 patients (Figure 1).

### Targeted MPS

DNA libraries for MPS on the Illumina MiSeq were prepared from 23–49 ng of input DNA. Capture enrichment was designed to target highly heterozygous SNPs across the dystrophin gene region (Chr X: 31,037,731‐33,457,670). Six to nine samples (equivalent to two to three patients) were multiplexed per sequencing run using 2 × 80 cycles paired‐end settings. More details can be found in Appendix B in the supporting information. Bioinformatics analysis included quality trimming of reads, alignment to genome build hg19, removal of duplicates and variant calling to obtain SNP counts (Appendix C in the supporting information).

### RHDO analysis for X‐linked disorders

**Figure 2:**
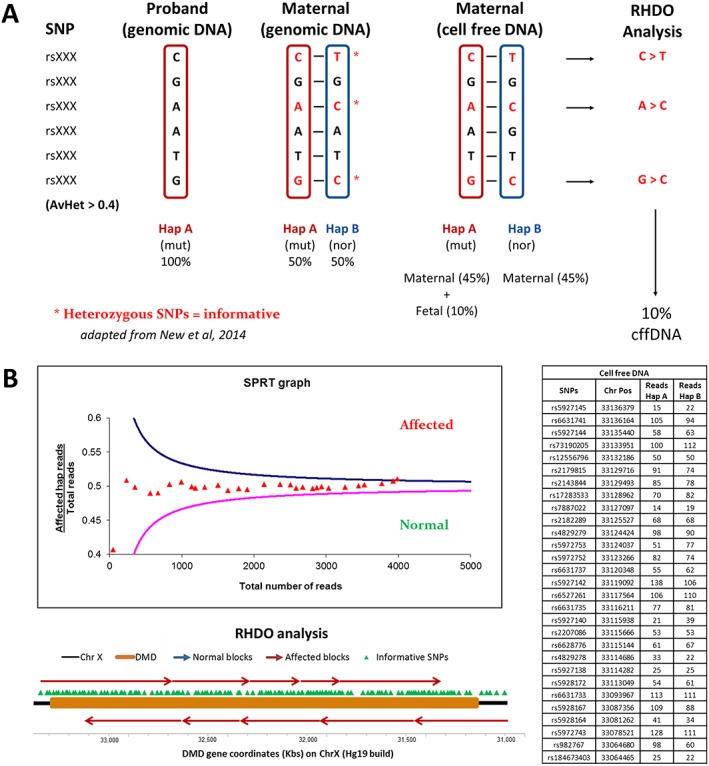
(A) The diagram summarises what is required to conduct relative haplotype dosage (RHDO) analysis for non‐invasive prenatal diagnosis of X‐linked disorders. The cumulative sequencing counts of SNP alleles are used to determine the proband and maternal haplotypes. SNPs that are heterozygous in the mother are informative and are used in the RHDO analysis. The allelic imbalance between the two haplotypes in the maternal cell‐free DNA (cfDNA) is used to calculate the fetal fraction (which has been set at 10% in this case). In this diagram, haplotype A is over‐represented in the maternal cfDNA, indicating that it has been inherited by the fetus. (B) SPRTs are used in RHDO analysis to determine the statistical significance of the allelic imbalance within a haplotype block. Cumulative sequencing counts of SNP alleles from plasma cfDNA (right table) are fed into the SPRT in order of chromosome position until a classification is made. Haplotype blocks are then plotted onto the dystrophin gene and provide the final outcome of the test (diagram). RHDO analysis is conducted from 5′ to 3′ and 3′ to 5′ in order to include all informative SNPs in the analysis and to better estimate the position of recombination sites

Relative haplotype dosage analysis measures the allelic imbalance between two haplotypes in plasma cfDNA to determine which haplotype has been inherited by the fetus.^19^, ^20^, ^24^ Haplotype phasing is conducted through sequencing of SNPs. In this study, RHDO analysis was adapted to disorders with an X‐linked mode of inheritance. When targeting an autosomal region, haplotyping of maternal, paternal and proband DNA is required to predict fetal inheritance.^24^ With X‐linked regions, however, paternal haplotyping is not necessary. In the case of DMD/BMD, the male proband DNA provides the haplotype linked with the mutated dystrophin gene (i.e. the affected haplotype), while the maternal DNA is needed to identify the informative heterozygous SNPs (Figure 2A). RHDO analysis uses sequential probability ratio tests to determine the allelic imbalance from the sequencing counts of informative SNP alleles obtained from plasma cfDNA^24^ (Figure 2B). The same counts are also used to determine the fetal fraction. Each sequential probability ratio tests classification is represented by a haplotype block and represents a statistically independent result determining the fetal inheritance of the region covered (Figure 2B). By identifying all the haplotype blocks across the region containing the dystrophin gene, fetal inheritance of the affected haplotype can be determined with a high level of resolution in a linkage‐based manner. In the case of a recombination event, which has up to 12% chance of occurring across the dystrophin gene,^31^ the haplotype blocks switch inheritance pattern after encountering the recombination site. Because the RHDO analysis is conducted in both directions (i.e. 5′ to 3′ and 3′ to 5′), the region in which the recombination site is positioned can be determined with high accuracy. Therefore, an accurate diagnosis can still be achieved in most of these cases by knowing the position of the DMD/BMD mutation carried by the patient. Data quality filters and RHDO analysis parameters were adopted from previous publications^20^, ^24^ (Appendix D of the supporting information). Fetal fraction was calculated using sequencing counts from plasma cfDNA (Appendix E of the supporting information).

### MLPA and linkage analysis

Routine invasive prenatal diagnosis of DMD/BMD patients was conducted using multiplex ligation‐dependent probe amplification (MLPA) analysis of the dystrophin gene (MRC‐Holland kits P034‐A2 and P035‐A2) to detect exon deletions/duplications. Linkage analysis was performed using a multiplexed set of fluorescent linkage markers (Appendix F of the Supporting Information and Table S1).

## Results

### Designing a highly efficient DMD custom probe library

**Figure 3:**
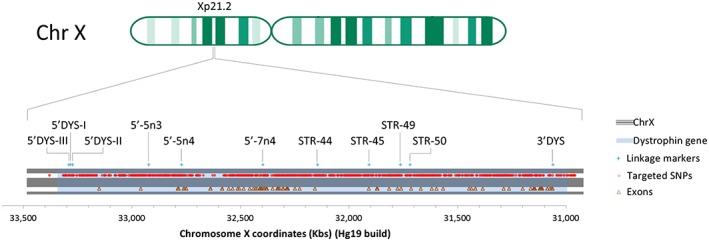
Diagram of the Xp21.2 locus on chromosome X containing the dystrophin gene, represented by the light blue highlighted area. The red dots indicate the chromosome position of SNPs with AvHet >0.4, which were targeted through capture‐based DNA library enrichment for non‐invasive prenatal diagnosis of Duchenne and Becker muscular dystrophies. The brown triangles indicate the chromosome position of all 79 exons contained in the dystrophin gene. The light blue crosses indicate the position of the markers routinely used in our laboratory for linkage analysis in Duchenne and Becker muscular dystrophies families

DNA library preparation for MPS was obtained by capture‐based targeted enrichment. This method has been successfully used for similar copy number variation tests in combination with RHDO analysis.^19^, ^20^ The probe library was designed to efficiently target 1350 SNPs with high average heterozygosity across a 2.4 Mb long region containing the dystrophin gene (ChrX: 31,037,731‐33,457,670) (Figure 3). This ensured a 40–50% probability that each targeted SNP would be informative. The highly efficient design allowed us to obtain ≈300–450 informative SNPs (Table 1) in a small captured area (201 Kb). It also ensured an even coverage of SNPs across the region of interest, which includes the chromosome positions of the markers that our laboratory routinely uses for linkage analysis of DMD/BMD (Figure 3).

**Table 1:** Summary of tests conducted on patients from groups 1 and 2

Family	Group	Mutation	Outcome	Gestation	Fetal fraction (%)	PND outcome	Reference haplotype	Informative SNPs used	Haplotype blocks (forward / reverse)	Classification accuracy (%)	Average sequencing depth of informative SNPs used
A	1	NA	HapA	13 w + 3 d	5.27	NA	CVS	422	15/14	100	160
B	1	NA	HapA	11 w + 6 d	15.19	NA	CVS	383	15/15	100	193
C	1	NA	HapA	12 w + 5 d	18.05	NA	CVS	365	15/15	100	52
D	1	NA	HapA	13 w + 2 d	5.83	NA	CVS	286	10/10	100	114
E	1	NA	HapA	13 w	26.62	NA	CVS	441	17/17	100	59
F	1	NA	HapA	11 w + 4 d	3.55	NA	CVS	441	15/15	90	247
G	1	NA	HapA	13 w + 3 d	14.84	NA	CVS	337	13/13	100	53
H	2	Del ex45	Affected	8 w + 4 d	9.48	Affected	Affected son	325	12/11	100	48
I	2	Del ex43	Unaffected	12 + 3 d	13.00	Unaffected	Affected brother	318	7/7	100	11

Prenatal diagnosis was conducted by invasive means on group 2 patients. The informative SNPs used represent SNPs which are heterozygous in the mother and comply with RHDO parameters (appendix D, supporting information). The numbers of haplotype blocks identified in the forward and reverse RHDO analysis are kept separate. The classification accuracy represents the percentage of haplotype blocks which showed an expected inheritance pattern. The average sequencing depth has been calculated on the informative SNPs used for the RHDO analysis.

### Results for group 1 patients: families A–G

Initial testing for the validation of our method was conducted on patients recruited to group 1. Eight patients were tested overall, and details on outcomes and testing parameters for seven of these patients are summarised in Table 1. Because of a technical issue, testing in one of these patients did not meet the minimum data quality criteria and has not been included (Appendix G of the Supporting Information and Table S2). Blood samples from all patients were taken between 11 and 14 weeks of gestation and extracted cfDNA showed varying fetal fractions ranging between 3–27%. On average, 536 [interquartile range (IQR) = 106] informative SNPs were identified for each patient, and 385 (IQR = 72) of these were used for RHDO analysis after quality filtering. The quantity of haplotype blocks classified (both for forward and reverse analyses) ranged between 10 and 17 depending on the number and sequencing depth of informative SNPs identified and the level of fetal fraction measured. The accuracy of haplotype block classifications was 100% in all cases bar one, in which three out of 30 haplotype blocks (two out of 15 in the forward analysis and one out of 15 in the reverse analysis) were incorrectly classified. This decrease in accuracy is probably due to the lower than 4% fetal fraction measured, which is often considered as the limit of sensitivity for non‐invasive prenatal tests.^32^, ^33^, ^34^ Overall, the method demonstrated a high consistency with all outcomes for group 1 patients resulting in the expected ‘haplotype A’.

### Results for group 2 patients: family H

**Figure 4:**
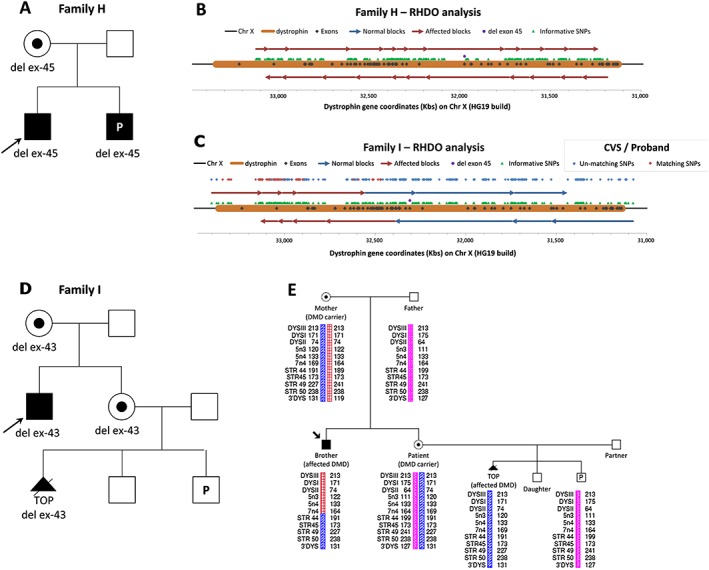
(A) Family tree of family H. (B) Diagram of relative haplotype dosage (RHDO) analysis results for family H, showing the chromosome position of the dystrophin gene, its exons, the informative SNPs identified and the mutation. Haplotype blocks are represented as red and blue arrows depending on whether they show an overrepresentation (red) or under‐representation (blue) of the affected haplotype. (C) Diagram of RHDO analysis results for family I. The switch from over‐representation to under‐representation of the affected haplotype indicates that a recombination event has taken place. The comparison between SNP alleles sequenced from the chorionic villus sampling (CVS) and the proband DNA samples is represented in the diagram by blue dots where unmatching alleles were observed and by red dots where matching alleles were found. (D) Family tree of family I. (E) Linkage analysis on family members of family I using Duchenne muscular dystrophy (DMD) markers. The marker name and size for both alleles are shown along the dystrophin gene region under each family member tested

In family H, the patient was a carrier of a deletion of exon 45 in the dystrophin gene, which is associated with DMD. She had had a previous affected child and was 8 weeks and 4 days pregnant at blood draw (Figure 4A). Genomic DNA from the affected boy was used to determine the affected haplotype. The fetal fraction of plasma cfDNA was measured at 9.24%, and RHDO analysis identified 23 haplotype blocks (12 in the forward analysis and 11 in the reverse) (Figure 4B). All haplotype blocks were classified as affected, indicating that the fetus had inherited the mutated dystrophin gene from the mother. MLPA analysis on the invasively obtained CVS sample confirmed the outcome. Testing parameters for this patient are summarised in Table 1.

### Results for group 2: family I

The patient in family I was a carrier of an exon 43 deletion, consistent with DMD. The brother of the patient was affected, and his genomic DNA was used to determine the affected haplotype (Figure 4D). The patient's gestational age was 12 weeks and 3 days at blood draw. Prior to the current pregnancy, she had had a healthy baby girl and a termination of pregnancy due to a positive diagnosis of DMD in a male fetus. Testing for this patient also included the genomic DNA extracted from the CVS as a further control. Because of a technical issue (Appendix G of the supporting information), our method performed poorly in this instance, showing a lower sequencing depth than we would usually consider acceptable (Table 1). However, the fetal fraction calculated using the haplotype obtained from the CVS sample showed 13% of cffDNA, and the RHDO analysis was able to identify 14 haplotype blocks overall (seven for the forward analysis and seven for the reverse) (Figure 4C). Therefore, we are presenting this case for the interesting implications it holds but would not have considered the test as viable in clinical practice. The initial fetal fraction calculation, performed using the haplotype from the affected brother as reference, yielded an extremely low amount of 0.63%. Knowing that the fetal fraction measured using the CVS as reference haplotype was 13%, we hypothesized that a switch in allelic imbalance must have occurred half way down the targeted region of interest, thus affecting the fetal fraction calculation. When we conducted the RHDO analysis using a fetal fraction of 13%, we indeed observed a single recombination event, which had taken place between chromosome coordinates 32,549,862 and 32,388,364. Given that the deletion of exon 43 would occur between chromosome coordinates 32,328,198 and 32,235,181, we predicted the outcome to be unaffected. This was confirmed by MLPA analysis following invasive testing. We then compared the haplotypes of the affected brother and of the CVS side by side to confirm the position of the recombination event. The red and blue dots in Figure 4C represent matching (red) and unmatching (blue) alleles of informative SNPs between the two haplotypes. As expected, unmatching alleles were observed across the whole area covered by haplotype blocks classified as unaffected (blue). However, we observed a considerable number of unmatching alleles (blue) within the region covered by the haplotype blocks classified as affected (red), where we expected to see only matching alleles (red). This suggested that the recombination event had not occurred in the fetus but in the brother of the patient. To confirm this, we conducted a linkage analysis on the family using markers routinely used in our laboratory (Figure 4E). The results confirmed that the recombination event had occurred in the brother. The DNA from the CVS of the patient's previous affected fetus was used as an additional positive control.

## Discussion

Non‐invasive prenatal testing is rapidly being implemented in many clinical genetics laboratories across the world. Fetal sexing and RhD typing using cffDNA are now in routine clinical service,^2^ while aneuploidy screening has been developed by several US‐based companies^13^, ^35^, ^36^, ^37^ and is now rapidly being introduced in public health services as well.^38^ In the UK, bespoke NIPD tests for exclusion of paternally inherited and *de novo* mutations have recently been developed through the RAPID project^10^, ^11^ and are now offered as a clinical service.^2^ Although technically possible, little attention has been given to NIPD of SGDs, mostly due to the limited number of patients who would request it and the prohibitively high‐testing costs.^2^, ^17^ Therefore, the NIPSIGEN project conducted at Birmingham Women's NHS Foundation Trust (UK) was funded to develop a method capable of delivering accurate NIPD for SGDs at a viable cost for the NHS. The newly developed method presented in this paper is capable of accurately testing patients at risk of carrying a male fetus affected with DMD/BMD, albeit at the condition of a DNA sample from the proband being available. Preliminary data presented in this paper have shown an overall accuracy in correctly classifying haplotype blocks of 100% (206/206) in patients with fetal fraction >4% and of 98.7% (233/236) overall. This underlines the reliability of the method, as each haplotype block represents a statistically independent result. The method was also able to detect a recombination event with high precision. This is a fundamental requirement for DMD/BMD testing, as the recombination rate across the whole dystrophin gene can be as high as 12%.^31^ By accurately assessing the position of the recombination site, our method enables the delivery of a correct diagnosis when the position of the maternal mutation on the dystrophin gene is known. However, the test would result as inconclusive if the DMD/BMD mutation is positioned within the area containing the recombination site. Results obtained from RHDO analysis on three additional patients (one from group 1 and two from group 2), which have not been presented due to the low quality in sequencing data, have also shown correct outcomes, thus underlining the robustness of the method (Appendix G of the supporting information and Table S2). Nevertheless, further improvements are necessary. In testing the patient from family I, we realised that fetal fraction cannot be accurately calculated by using the allelic imbalance measured on a region of chromosome X in the presence of a recombination. Additionally, family linkage analysis might be necessary when having to use the DNA of a patient's affected brother, or other male relative, to identify the affected haplotype in order to rule out the possibility of previous recombination events. However, this would not be required when using the DNA of a patient's previous affected child. It would also not be necessary if the patient's father DNA is used for the identification of the affected haplotype, which might be the case in patients carrying a BMD mutation. Additionally, in cases of both DMD and BMD where DNA from an affected family member is not available, the DNA of the patient's unaffected father or previous healthy son can be used for the identification of the unaffected haplotype, in which case RHDO analysis would determine the over‐representation or under‐representation of the unaffected haplotype instead of the affected one. However, when identifying the unaffected haplotype from the patient's father to use for RHDO analysis, the DMD/BMD carrier status of both the patient and her mother should be confirmed, as this option would not be appropriate for pregnancies where the pregnant mother is a carrier of an apparently *de novo* DMD/BMD mutation. Finally, it is important to note that this method is subject to the common limitations linked with cffDNA analysis, such as the impossibility of obtaining a viable diagnostic result in the case of twin pregnancies; in the presence of a vanishing twin; in the presence of maternal somatic mosaicism; or if the patient has undergone transplant surgery (ie the patient has been transplanted with a donor organ).

In order to address some of the issues listed earlier, we plan to further improve our method by designing a new probe library. Coverage of a number of SNPs across various autosomes will be included for the accurate measurement of fetal fraction. Additional SNPs will be targeted at the 3′ and 5′ untranslated regions of the dystrophin gene to take into account the possibility of fewer informative SNPs being identified due to a pregnancy between consanguineous parents or to the presence of a large deletion. Taken together, these advances will significantly improve the test accuracy and applicability.

At present the main drawback to the implementation of NIPD for SGDs in public health services has been the elevated cost incurred.^20^, ^22^ In addition, the number of patients who would benefit from these tests is small, and therefore, costs cannot be significantly reduced by increasing the multiplexing capacity of the test. Our method addresses both these issues by using the lower cost Illumina MiSeq sequencing platform and allowing a multiplexing capacity of up to three patients for each sequencing run. Additionally, testing of maternal plasma is performed alongside the proband and maternal genomic DNA, thus considerably reducing additional haplotyping costs. Taking these considerations into account, we have calculated the laboratory cost of our NIPD test for DMD/BMD to be £650 per patient (consumables and staff costs only) when multiplexing three patients on one sequencing run. However, the full cost will also need to take into account equipment costs and other overheads. As a full economic analysis has not yet been performed, we are unable to comment on how this would compare with current clinical practice. Nevertheless, the decrease in sequencing costs and the increase in requests by patients at risk who would not consider invasive testing,^39^ bode well for the future implementation of our method within a clinical setting. Use of the Illumina NextSeq would also allow for an increased multiplexing capacity of four to eight patients, which would further reduce testing costs. Running the test on a weekly basis, the turn‐around‐time would stand at seven to ten working days depending on the day of the week the blood sample is received. This would allow patients to receive a diagnosis within the first trimester of pregnancy, as the blood sample can be taken from as early as 8 weeks of gestation. Our NIPD test will therefore deliver significant clinical benefits in comparison with the current clinical pathway, as it will provide patients with more time to manage their pregnancy while also eliminating the risk of miscarriage associated with invasive procedures.

The successful development of an NIPD test for patients at risk of carrying a fetus affected with DMD/BMD suggests that the same method can potentially by applied to many other SGDs. Indeed, the different types and locations of mutations associated with SGDs do not affect RHDO analysis. On this premise, we are adapting our method to include other disorders such as spinal muscular atrophy, CAH and cystic fibrosis. This will increase our NIPD testing repertoire and the number of patients we can offer this service to, which will prove beneficial in ensuring a quick turn‐around‐time and maintaining testing costs at the lowest possible level (by grouping together at least three patients a week per sequencing run).

To conclude, RHDO analysis has been successfully used to determine the genome‐wide genetic and mutational profile of the fetus^24^ and for NIPD of β‐thalassemia^19^ and CAH,^20^ albeit at a prohibitive cost. For the first time, we have proven that NIPD of SGDs can be performed in a clinical setting through the use of highly efficient targeted MPS and RHDO analysis. Preliminary data presented in this paper have shown that our NIPD test for DMD/BMD was accurate in patients with a fetal fraction higher than 4%. In the near future, we aim to improve our method and adapt it to other SGDs in order to offer NIPD for a wide panel of disorders to our patients.

## Supporting information

Supporting info item
