# Supplementary material for: Non‐invasive prenatal diagnosis of Duchenne and Becker muscular dystrophies by relative haplotype dosage
Source: Prenat Diagn. 2016 Feb 23;36(4):312–20. doi: 10.1002/pd.4781 (PMC4864947; doi:10.1002/pd.4781)
Supplement: Supplementary file 1 — Supporting info item [file PD-36-312-s001.docx]

**SUPPLEMENTARY MATERIALS**

**Appendix A: sample collection and processing**

Patient recruitment was conducted through the NIPSIGEN study (“NIPSIGEN: clinical translation of NIPD for single gene disorders”; REC approval number: 13/NW/0580). Pregnant women referred to West Midlands Regional Genetics Laboratory for increased risk of aneuploidies were recruited to group 1; pregnant women who carried a DMD mutation were recruited at a national level (UK) to group 2. All patients were requested to donate 20 ml of peripheral blood prior to CVS (see table 1 for details of gestational age at blood draw) which was collected in EDTA tubes for group 1 patients and Cell-Free DNA BCT tubes (Streck) for group 2. Plasma isolation was conducted within 24 hours of bleeding for EDTA tubes and 76 hours for Cell-Free DNA BCT tubes. Blood samples were centrifuged at 1600g for 10 min; isolated plasma was further centrifuged at 16,000g and stored at -80˚C. The blood cell portion was stored at -20˚C. CfDNA was extracted from 4 ml of plasma on the automated QIAsymphony platform (Qiagen) using the QIAsymphony DSP Virus/Pathogen Midi kit and following the custom modified protocol for DNA extraction from large volumes of sample. DNA was eluted in a final volume of 60 µl. Maternal genomic DNA was extracted from the leukocytes contained in 1 ml of the blood cell portion (post plasma isolation) using the QIAamp MinElute Virus Spin kit on the automated QIAcube platform (Qiagen) following the supplier’s protocol. Genomic DNA from CVS samples was extracted with the EZ1 DNA Tissue kit on the automated EZ1 platform (Qiagen). Extracted DNA concentration was measured using the Qubit 2.0 High Sensitivity kit (Invitrogen).

**Appendix B: Targeted MPS**

Genomic DNA from maternal, proband and CVS samples was sheared to a length of 180-220 bp on the Covaris M220. CfDNA does not require shearing as it is already fragmented in nature ^1^. 23-49 ng of input DNA per sample was used to prepare libraries for sequencing on the Illumina MiSeq using the KAPA Library Preparation kit for Illumina platforms (KAPA biosciences). Targeted capture enrichment was performed using a custom SeqCap EZ Choice probe library (Nimblegen) to enrich for DNA fragments containing SNPs with average heterozygosity > 0.4 across the dystrophin gene region (Chr X: 31,037,731-33,457,670; hg19 build). The total captured area was 201 Kb at 80% probe coverage. 6-9 samples (equivalent to 2-3 patients) were multiplexed per sequencing run on the MiSeq (Illumina) using the 150 cycle PE V3 kit. The settings used included single indexing and 2x80 cycles paired-end sequencing.

**Appendix C: Bioinformatics pipeline**

FASTQ files obtained from the MiSeq were quality trimmed using Trimmomatic v0.32 ^2^ to remove reads that fall below a quality score threshold of 30. Reads were then aligned to the human genome, hg19, with bowtie2 v2.1.0 ^3^. Alignment files were pre-processed and duplicates removed using a combination of Picard tools v1.97 ^4^, SAMtools v0.1.19 ^5^ and GenomeAnalysisTK v2.7-4-g6f46d11^6–8^. Platypus v0.6.0 variant caller^9^ was used to obtain counts for every SNP contained in the region of interest.

**Appendix D: RHDO analysis parameters**

RHDO analysis was adapted from previous publications ^10,11^ to account for recessive X-linked inherited disorders, where heterozygous SNPs in the mother are the only type of informative SNPs. This is due to the fact that only male pregnancies are tested for these disorders and the single X chromosome carried by the fetus is solely responsible for the allelic imbalance detected in the cfDNA extracted from maternal plasma. Phasing of maternal and proband (or CVS) haplotypes was calculated using Excel worksheets (Microsoft Office 2010). Informative SNPs were required to show a sequencing depth ≥ 30 (in genomic DNA and cfDNA samples) and be separated from each other by ≥ 200 bp, in order to reduce bias ^11^. Haplotype blocks had to contain ≥ 25 informative SNPs, to minimize stochastic influences. For a recombination event to be called, two or more consecutive haplotype blocks showing a switch in fetal inheritance had to be observed ^10^. An odds ratio of 1200 was used in SPRT classifications ^10^. SPRT calculations and graphical representations of RHDO analysis outcomes were obtained using Excel worksheets.

**Appendix E: Calculation of fetal fraction in plasma cfDNA**

Fetal fraction was calculated on the cumulative haplotype imbalance observed in plasma cfDNA. Cumulative sequencing counts from informative SNPs for haplotype A (cHapA) and haplotype B (cHapB) were used in the following formula in order to calculate the fetal fraction:

cffDNA % = [(|cHapA – cHapB| * 2) / (cHapA + cHapB + |cHapA – cHapB|)] * 100

**Appendix F: DMD/BMD linkage markers**

The markers for DMD/BMD linkage analysis are routinely used in our laboratory for prenatal diagnostic testing and include: DYS III, DYS I, DYS II, 5’-5n3, 5’-5n4, 5’-7n4, STR 44, STR 45, STR 49, STR 50, 3’DYS. Multiplexed sets of these fluorescent linkage markers were run on an ABI3130XL genetic analyser. Table S1 lists the coordinates of the markers on chromosome X, hg19 build.

**Appendix G: Technical issues and additional results: families J - L**

During validation, we encountered a technical issue with our method which resulted in uncharacteristically low sequencing depths for four patients tested together in a single run (one of these patients corresponds to family I and is presented in the results section for the interesting implications it holds). This was caused by the degradation of sequencing adapters (due to repeated freeze/thaw cycles), which resulted in reduced adapter ligation during the DNA library preparation step and, consequentially, poor library yields and decreased sequencing depth. As informative SNPs need to show a sequencing depth ≥ 30 to pass data quality check, we classified these results as “failed”. Nevertheless, when applying RHDO analysis to this data (after having lowered the sequencing depth cut-off limit to 20 for the genomic DNA samples and to 0 for the plasma cfDNA) we found that the final results showed 95.33% accuracy of haplotype block classifications (table S2). All results correlated with expected outcomes determined by invasive testing. The identified technical issue was resolved by requiring that a fresh aliquot of sequencing adapters be prepared immediately prior to the DNA library preparation step. No further issues were identified in our method.

**Table S1** List of markers routinely used in our laboratory for DMD/BMD linkage analysis with associated chromosome coordinates (hg19 build).

| **DMD/BMD linkage markers** | | |
| --- | --- | --- |
| **Markers** | **Chromosome coordinates** | |
|  | **5' start** | **3' end** |
| DYS_III | 33,369,580 | 33,369,794 |
| DYS_I | 33,361,363 | 33,361,538 |
| DYS_II | 33,351,784 | 33,351,857 |
| 5'-5n3 | 33,001,696 | 33,001,815 |
| 5'-5n4 | 32,849,715 | 32,849,868 |
| 5'-7n4 | 32,474,615 | 32,474,782 |
| STR44 | 32,223,036 | 32,223,218 |
| STR45 | 31,985,385 | 31,985,559 |
| STR49 | 31,840,838 | 31,841,071 |
| STR50 | 31,797,669 | 31,797,911 |
| 3-DYS | 31,139,622 | 31,139,755 |

**Table S2** Summary of tests that have “failed” due to sub-standard quality parameters. Prenatal diagnosis (PND) was conducted by invasive means on group 2 patients. The informative SNPs used represent SNPs which are heterozygous in the mother and comply with RHDO parameters (appendix D, supplementary material). The numbers of haplotype blocks identified in the forward and reverse RHDO analysis are kept separate. The classification accuracy represents the percentage of haplotype blocks which showed an expected inheritance pattern. The average sequencing depth has been calculated on the informative SNPs used for the RHDO analysis.

| **Family** | **Group** | **Mutation** | **Outcome** | **Gestation** | **Fetal fraction**  **(%)** | **PND outcome** | **Reference haplotype** | **Informative SNPs used** | **Haplotype blocks (forward / reverse)** | **Classification accuracy** | **Average sequencing depth of informative SNPs used** |
| --- | --- | --- | --- | --- | --- | --- | --- | --- | --- | --- | --- |
| J | 1 | NA | HapA | 13 w + 1 d | 17.20 | NA | CVS | 286 | 4 / 4 | 100% | 4 |
| K | 2 | c.6901C>T (ex47) | Affected | 9 w + | 11.78 | Affected | Affected son | 258 | 3 / 4 | 86% | 8 |
| L | 2 | Dup ex2 | Affected | 11 w + 6 d | 9.98 | Affected | Affected son | 275 | 3 / 3 | 100% | 9 |

**References**

1 Chan KCA. Size Distributions of Maternal and Fetal DNA in Maternal Plasma. *Clin Chem* 2004; **50**: 88–92.

2 Bolger AM, Lohse M, Usadel B. Trimmomatic: a flexible trimmer for Illumina sequence data. *Bioinforma Oxf Engl* 2014; **30**: 2114–2120.

3 Langmead B, Salzberg SL. Fast gapped-read alignment with Bowtie 2. *Nat Methods* 2012; **9**: 357–359.

4 Picard. http://broadinstitute.github.io/picard/ (accessed 28 Aug2015).

5 Li H, Handsaker B, Wysoker A, Fennell T, Ruan J, Homer N *et al.* The Sequence Alignment/Map format and SAMtools. *Bioinformatics* 2009; **25**: 2078–2079.

6 McKenna A, Hanna M, Banks E, Sivachenko A, Cibulskis K, Kernytsky A *et al.* The Genome Analysis Toolkit: a MapReduce framework for analyzing next-generation DNA sequencing data. *Genome Res* 2010; **20**: 1297–1303.

7 DePristo MA, Banks E, Poplin R, Garimella KV, Maguire JR, Hartl C *et al.* A framework for variation discovery and genotyping using next-generation DNA sequencing data. *Nat Genet* 2011; **43**: 491–498.

8 Van der Auwera GA, Carneiro MO, Hartl C, Poplin R, Del Angel G, Levy-Moonshine A *et al.* From FastQ data to high confidence variant calls: the Genome Analysis Toolkit best practices pipeline. *Curr Protoc Bioinforma Ed Board Andreas Baxevanis Al* 2013; **11**: 11.10.1–11.10.33.

9 Rimmer A, Phan H, Mathieson I, Iqbal Z, Twigg SRF, WGS500 Consortium *et al.* Integrating mapping-, assembly- and haplotype-based approaches for calling variants in clinical sequencing applications. *Nat Genet* 2014; **46**: 912–918.

10 Lo YMD, Chan KCA, Sun H, Chen EZ, Jiang P, Lun FMF *et al.* Maternal Plasma DNA Sequencing Reveals the Genome-Wide Genetic and Mutational Profile of the Fetus. *Sci Transl Med* 2010; **2**: 61ra91–61ra91.

11 New MI, Tong YK, Yuen T, Jiang P, Pina C, Chan KCA *et al.* Noninvasive prenatal diagnosis of congenital adrenal hyperplasia using cell-free fetal DNA in maternal plasma. *J Clin Endocrinol Metab* 2014; **99**: E1022–1030.
